# Aortic Root Abscess Presenting as Pyrexia of Unknown Origin and the Importance of Echocardiography

**DOI:** 10.1155/2013/636519

**Published:** 2013-05-23

**Authors:** Prashanth Panduranga

**Affiliations:** Department of Cardiology, Royal Hospital, PB 1331, 111 Muscat, Oman

## Abstract

Aortic root abscess in patients with aortic endocarditis is not uncommon. Aortic root abscess may cause persistent sepsis, worsening heart failure, conduction abnormalities, fistula formation, and an increased need for surgery. We present a young patient with aortic root abscess presenting as pyrexia of unknown origin. She had acute severe aortic and mitral regurgitation which produced very soft murmurs that were easily missed. This report reiterates that a high index of suspicion is needed in suspecting valvular endocarditis as well as a comprehensive transthoracic and transesophageal echocardiographic examination to diagnose complications like aortic root abscess.

## 1. Introduction

Aortic root abscess in patients with aortic endocarditis is not uncommon. Aortic root abscess may cause persistent sepsis, heart failure, conduction abnormalities, fistula formation, and an increased need for surgery. We present a young patient with aortic root abscess presenting as pyrexia of unknown origin that was diagnosed by echocardiography.

## 2. Case 

**Figure 1:**
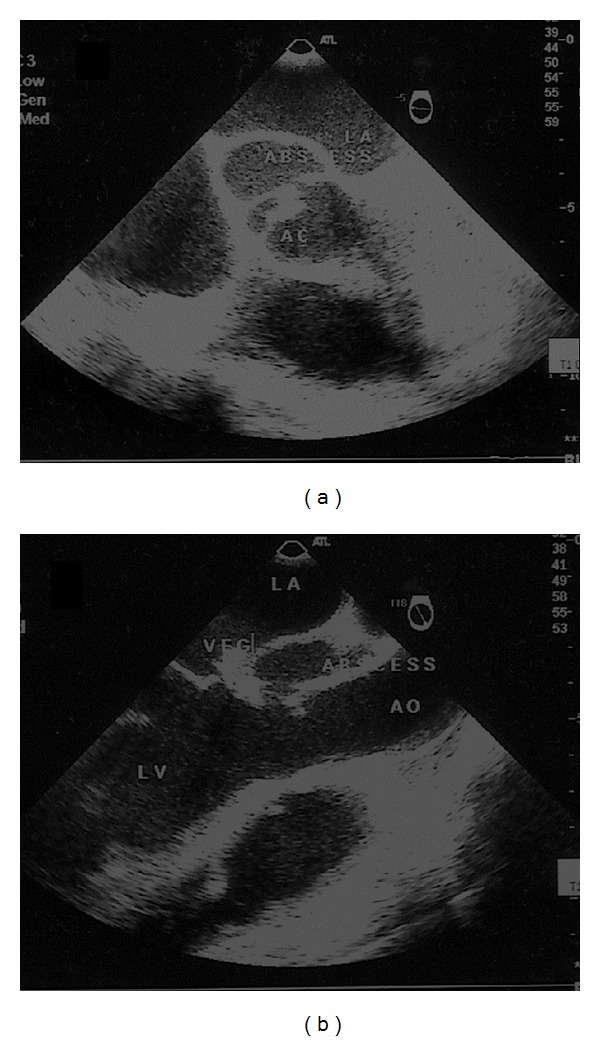
(a) Midesophageal-aortic valve short-axis view showing aortic root abscess (arrowheads) in relation to noncoronary cusp. (b) Midesophageal-LV long-axis view showing vegetation (VEG) on the base of anterior mitral leaflet with aortic root abscess (ABSCESS). LV: left ventricle; LA: left atrium; AO: aorta; VEG: vegetation; ABSCESS: aortic root abscess.

**Figure 2:**
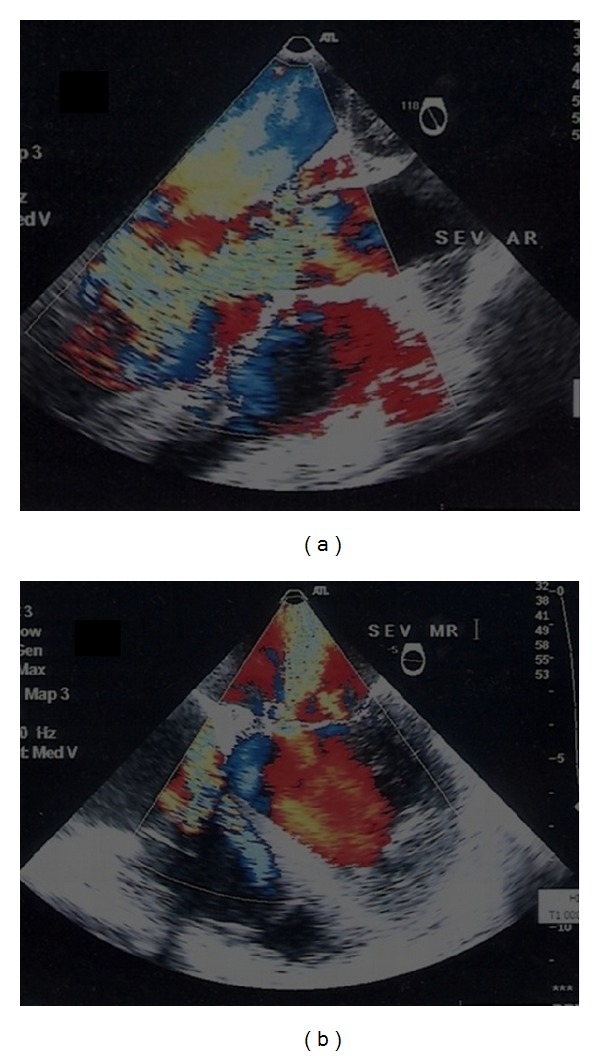
Midesophageal-LV long-axis view (a) and four-chamber view (b) showing severe aortic and mitral regurgitation, respectively.

A 26-year-old female with no past medical problems was admitted to critical care unit of our hospital with history of intermittent high-grade fever (39-40°C) for 3-week duration. She was extensively investigated in other hospitals with results yielding negative blood culture, autoimmune profile, and immunodeficiency profile. She was diagnosed to have pyrexia and sepsis of unknown origin. She had received multiple antibiotics during this period from other hospitals. Apparently previous cardiac examination was normal. Clinically she was febrile, tachycardic, tachypneic, and hypotensive. Chest X-ray showed pulmonary edema. Careful clinical examination revealed elevated jugular venous pressure, soft aortic early diastolic murmur, and a soft pan systolic murmur at apex. ECG showed sinus rhythm with normal PR interval. An urgent bedside transthoracic echocardiogram (TTE) revealed dilated left ventricle, large vegetation attached to base of anterior mitral leaflet with severe mitral and aortic regurgitation with EF 45%. The aortic valve looked edematous, but no clear-cut aortic root abscess was seen. The valves were thickened suggesting rheumatic etiology. Laboratory investigations revealed anemia, leucocytosis with markedly elevated inflammatory markers. She was immediately shifted to OR, and a transesophageal echocardiogram (TEE) confirmed TTE findings. In addition there was an aortic root abscess behind the noncoronary cusp (Figure 1) along with perforation of anterior mitral leaflet with severe aortic and mitral regurgitation (Figure 2). She underwent successful pericardial patch closure of the aortic abscess cavity and double valve replacement. There was no mitral abscess or fistulae noted intraoperatively. Histopathology of explanted mitral and aortic valve tissue showed fibrinoid necrosis and exudates with acute inflammatory cells surrounding colonies of Gram-positive bacteria with both cocci and bacilli. Her blood cultures were reported negative. 

## 3. Discussion

In the International Collaboration on Endocarditis Merged Database (ICE-MD) study, among 311 patients who had definite aortic valve endocarditis, 22% had periannular abscesses [1]. Periannular abscesses were more likely in patients with prosthetic valve and coagulase-negative staphylococcal endocarditis, and those patients less likely to have streptococcal endocarditis than were patients who did not develop abscess. Patients who had abscess were more likely to undergo surgery (84% versus 36%), and their in-hospital mortality rate was higher (19% versus 11%). By multivariate analysis, systemic embolization, heart failure, and mortality did not differ between those who developed abscess and those who did not. However, *Staphylococcal aureus* infection was found to be an independent prognostic factor for mortality in patients who have abscess formation. 

In a study, when compared with the transthoracic approach, TEE was associated with a significantly higher rate of abscess detection (80% versus 36%) especially for aortic root abscesses [2]. In another study, abscess could be diagnosed by peroperative TEE in 48% of patients only. Majority of the missed abscesses were localized on the posterior mitral annulus [3]. It is well known that acute severe valvular regurgitation is a surgical emergency, but properly timed diagnosis can be difficult especially in a patient without known cardiac disease as seen in this patient [4]. This is due to the fact that examination findings of acute regurgitation are subtle, with nonspecific clinical presentation, and the condition may be misdiagnosed as sepsis, pneumonia, or nonvalvular heart failure [4]. In addition, examination findings typically seen in chronic regurgitation like chamber enlargement are absent or subtle, and the murmurs are frequently soft. Furthermore, tachycardia and tachypnea further impair the detection of faint murmurs [4]. In a recent study, as compared with conventional treatment, early surgery in patients with infective endocarditis and large vegetations significantly reduced systemic embolism and death [5]. This report reiterates that a high index of suspicion is needed in suspecting valvular endocarditis with acute regurgitation in patients with sepsis of unknown cause as well as a comprehensive clinical and echocardiographic examination to diagnose complications like aortic root abscess.
